# Emotional abuse interacts with borderline personality in adolescent inpatients with binge-purging eating disorders

**DOI:** 10.1007/s40519-021-01142-3

**Published:** 2021-03-07

**Authors:** J. Spiegel, S. Arnold, H. Salbach, E. G. Gotti, E. Pfeiffer, U. Lehmkuhl, C. U. Correll, C. Jaite

**Affiliations:** 1 Department of Psychiatry, Psychotherapy and Psychosomatics, Vivantes Hospital Urban, Berlin, Germany; 2 Department of Child and Adolescent Psychiatry, Psychosomatic Medicine and Psychotherapy, Charité – Universitätsmedizin Berlin, corporate member of Freie Universität Berlin, Humboldt-Universität zu Berlin, and Berlin Institute of Health, Augustenburger Platz 1, 13353 Berlin, Germany; 3 Department of Clinical Psychology and Psychotherapy, Freie Universität Berlin, Berlin, Germany; 4 Department of Psychiatry, The Zucker Hillside Hospital, Northwell Health, Glen Oaks, NY USA; 5 Zucker School of Medicine at Hofstra/Northwell, Department of Psychiatry and Molecular Medicine, Hempstead, NY USA

**Keywords:** Bulimia nervosa, Anorexia nervosa binge-purging, Adolescents, Childhood emotional abuse, Borderline personality

## Abstract

**Purpose:**

Childhood abuse is associated with an increased risk of developing eating disorders (EDs) as well as personality disorders (PDs). However, their interaction is still uncertain, particularly in adolescents. This study investigates the correlations between childhood emotional neglect (CEN), childhood emotional abuse (CEA), and obsessive-compulsive and borderline personality styles in female adolescent inpatients with eating disorders (EDs).

**Methods:**

One hundred and twenty-eight inpatients (ages 14-18) were assessed, 54 were diagnosed with restricting-type anorexia nervosa (AN-R) and 33 with a binge-purging ED [BP-ED; comprising patients with binge-purging type anorexia nervosa (AN-BP), *n* = 15, and bulimia nervosa (BN), *n* = 18]. Fifty healthy participants made up the control group (CG). CEN and CEA were assessed with the Childhood Trauma Questionnaire, while the Personality Style and Disorder Inventory was implemented to determine personality styles.

**Results:**

A MANOVA revealed a significant main effect of CEA on spontaneous-borderline personality style [*F*(8,119) = 17.1, *p* < 0.001, *η*^*2*^ = 0.126], as well as a main effect of ED group on spontaneous-borderline [*F*(2,119) = 3.1, *p* = 0.048, *η*^*2*^ = 0.050]. A significant interaction between ED group, CEA, and spontaneous-borderline was found [*F*(2,119) = 3.5, *p* = 0.034, *η*^*2*^ = 0.055] with BP-ED showing significantly higher scores in CEA (9.3 ± 4.0) and in spontaneous-borderline (14.2 ± 6.2).

**Conclusions:**

Considering CEA and borderline personality style in adolescent inpatients with BN or AN-BP may help improve the understanding of the etiology and maintenance of BP-ED and provide more effective treatment targets.

**Level of evidence:**

Level III, case–control analytic study.

## Background

Previous studies suggest sexual and physical abuse as non-specific risk factors for the development of anorexia nervosa (AN) and bulimia nervosa (BN) [1–5]. However, childhood emotional neglect (CEN) and childhood emotional abuse (CEA) also seem to be of particular importance in AN and BN developments [6–12]. CEN is predominantly passive and can be described as a lack of emotional affection or appreciation for the child. CEA is characterized by permanent hostile rejection or devaluation [13]. For CEA, prevalence rates of 24% in restricting-type AN (AN-R), 48% in binge-purging type AN (AN-BP), and 81% in BN have been reported [6]. However, little is known about the occurrence of CEN in individuals with AN or BN. Preliminary findings of a meta-analysis [14] suggest higher CEN prevalence in patients with eating disorders (EDs) (53.3%) compared to the general population (18.4%). Since data in the meta-analysis were aggregated over all ED subgroups, it still remains unclear whether CEN rates differ significantly between said subgroups [14].

CEN and CEA are also associated with an increased risk of developing a personality disorder (PD) [15–17]. For example, Johnson et al. [16] found that CEA increased the risk for developing PDs. Personality styles and PDs seem to play a central role in the development and maintenance of AN and BN and have also been found to exacerbate prognosis and treatment response [18–23]. PDs are highly comorbid in AN and BN [24–27] with a mean proportion of 0.49 in AN and 0.54 in BN compared to 0.09 in healthy control participants [26]. PD comorbidity rates of 14.1% in AN-R, 58.8% in AN-BP, and 48.0% in BN were reported for adolescent patients [28]. While significantly higher rates of comorbid borderline PD have been found in BN (33%) and AN-BP patients (29.4%) [29] compared to AN-R patients (12%), obsessive-compulsive PD appears to be significantly more frequent in AN (0.23) than in BN (0.12) [26]. Overall, CEN and CEA are associated with a higher risk of developing AN or BN as well as PDs. The interaction between these factors in adolescents with AN and BN is still unknown. Concerning the etiology of borderline PD, various models have been discussed in the literature. Exposure to an invalidating, often traumatic environment is central to these theories and has been found to be a contributing factor to the development of borderline PD [30]. Difficulties in regulating emotions may account for the association between childhood abuse experiences and the development of borderline PDs [30, 31]. Thus, impulsive behaviors may serve as emotion regulation strategies in abused patients with EDs. However, there is no research regarding the link between CEN, CEA, and compulsive behaviors in this patient group, despite evidence that impulsive as well as compulsive behaviors can serve as emotion regulation strategies [32].

To our knowledge, there are no published studies investigating the interaction between CEN, CEA, and specific personality styles in adolescents with AN and BN. The interaction between these factors in adolescents with AN and BN is still unknown. Since childhood and adolescence are pivotal stages in the development of personality [33, 34], it seems crucial to conduct studies in young patients with EDs and thereby contribute to a better understanding of the development and maintenance of EDs.

Thus, this study aimed to examine the interaction between CEN or CEA and obsessive-compulsive or borderline personality styles in adolescent inpatients with binge-purging EDs (AN-BP and BN) compared with AN-R and a healthy control group (CG). As previous studies mainly dealt with physical and sexual abuse in ED patients, this study focused on CEN and CEA. Obsessive-compulsive and borderline PDs are the most frequent specific PD diagnoses in adolescents with EDs [27, 35]. Therefore, we focused our analysis on these two specific personality styles. It was hypothesized that traumatic childhood experiences and personality styles would differ between patients with binge-purging symptoms (AN-BP and BN), patients with AN-R, and a healthy CG. We expected an association between CEN, CEA, and borderline personality style in patients with binge-purging EDs (AN-BP and BN) as well as between CEN, CEA, and obsessive-compulsive style in patients with AN-R.

## Methods

### Participants and setting

A sample of 87 adolescent patients with EDs was recruited from a unit specializing in treating EDs at a child and adolescent psychiatry department. To be included in the study, patients had to be diagnosed with AN-R, AN-BP, or BN (AN-BP and BN will be subsequently grouped together as BP-ED) according to the International Statistical Classification of Diseases, 10th Version (ICD-10) [36], and be between 14 and 18 years old. An additional 50 healthy control participants of the same age range were recruited from local high schools. Exclusion criteria for the CG were the presence of any psychiatric disorder, including EDs. Participants and their legal guardians provided written informed consent prior to their participation. The CG received financial compensation for the time, expense, and effort to come for visits not related to clinical care as part of their study participation. Ethical approval was obtained through the ethics committee of the Charité – Universitätsmedizin Berlin.

### Measures

To objectively calculate the Body Mass Index (BMI) and its percentiles, the body height (cm) and body weight (kg) of all participants was recorded using a standardized scale and height measurement. ED diagnosis and general psychopathology were assessed according to ICD-10 by trained clinical psychologists and medical doctors. The absence of an ED or any other psychiatric diagnosis in the CG was ensured using the structured expert interviews *Structured Inventory for Anorexic and Bulimic Eating Disorders for DSM-IV and ICD-10* (SIAB) [37] and the *Composite International Diagnostic Interview (CIDI-DIA-X)* [38]. The SIAB [37] was implemented to assess the presence of an ED within the previous three months. The SIAB has good inter-rater reliability (*κ* = 0.81) and satisfactory convergent and discriminatory construct validity [37]. The CIDI-DIA-X is meant to confirm or refute the presence of any mental disorder [38] and was used to screen for psychiatric illnesses in CG participants. For most mental disorders, the CIDI-DIA-X inter-rater reliability falls between 0.97 and 1.0, while the inter-rater Kappa varies between 0.67 and 0.99 [39].

CEN and CEA were assessed retrospectively with the subscales of emotional neglect and emotional abuse from the German version of the *Childhood Trauma Questionnaire* (CTQ) [40]. The CTQ items were answered on a five-point Likert scale [“not at all” (1) to “very often” (5)]. CEN and CEA were assessed with five items each, such as “There was someone in my family who helped me feel that I was important or special” (CEN, reversed) or “People in my family called me things like ‘stupid,’ ‘lazy,’ or ‘ugly’” (CEA). The CTQ scales’ internal consistency was satisfactory with *α* ≥ 0.80. Construct validity was demonstrated by a positive correlation with anxiety and depression measures as well as a negative correlation with life satisfaction [40].

Using the self-report *Personality Style and Disorder Inventory* (PSDI) [41], the personality styles conscientious-compulsive and spontaneous-borderline were assessed. Each subscale contains ten items answered on a four-point Likert scale [“does not apply at all” (0) to “strongly agree” (3)]. The personality style conscientious-compulsive was assessed with, for instance, the item “I have firm principles that I always adhere to”. An example of an item assessing the spontaneous-borderline personality style is “My feelings about something or someone frequently change very abruptly”. Across all scales, the internal consistency varies between *α* = 0.73 and 0.85, and the test-retest ranges reliability between *r* = 0.68 and 0.83. The correlation with various psychological parameters, such as depression, psychosomatic symptoms, or the Big 5 [42], demonstrates a good construct validity [41].

### Statistical analysis

All statistical analyses were performed using the IBM Statistical Package for Social Sciences (SPSS) Version 25 [43]. A significance level of *α* = 0.05 was predefined for all statistical procedures. Preliminary analyses included calculating descriptive statistics for sample characteristics (age, body height, body weight, BMI, BMI percentiles, and comorbidities) for each group (AN-R, BP-ED, and CG). Differences in continuous variables between groups were analyzed with a one-way analysis of variance (ANOVA). Post hoc analyses were conducted using Tukey’s Honestly Significant Difference (HSD) test. Group differences in nominal variables were examined with a Chi-square test.

A two-way factorial multivariate analysis of variance (MANOVA) was conducted as primary analysis. In this analysis, the group was the fixed factor (AN-R, BP-ED, and CG), CEN and CEA were covariates, and *conscientious-compulsive* and *spontaneous-borderline* were dependent variables. Eta squared (*η2*) represents the percentage of variability in the dependent variable that can be accounted for by the independent variable. Significant effects are graphically displayed in a bar graph.

## Results

A total of 135 female adolescents participated in the study. Due to the presence of a psychiatric disorder, as assessed with the CIDI-DIA-X [39], 7 of the 50 subjects recruited for the CG group were not included in the analysis. Therefore, data from 128 participants were analyzed. 42.2% (*n* = 54) participants were diagnosed with AN-R, 25.8% with BP-ED (AN-BP: *n* = 15, BN: *n* = 18), and 32.0% (*n* = 41) did not fulfill the criteria of any psychiatric diagnosis (CG). Sample characteristics are presented in Tables 1 and 2.

**Table 1:** Sample characteristics: age and BMI

	Group			ANOVA			
	AN-R (*n* = 54) *M* ± SD	BP-ED (*n* = 33) *M* ± SD	CG *(n* = 41) *M* ± SD	*F*	*df*	*p*	Post hoc analysis
Age (in years)	16.0 ± 1.4	16.6 ± 1.5	15.8 ± 1.3	3.8	3	0.025*	BP-ED = AN-R; BP-ED > CG; AN-R = CG
BMI	15.1 ± 1.7	18.6 ± 2.6	20.7 ± 1.5	102.5	3	< 0.001*	CG > BP-ED > AN-R
BMI percentile	2.1 ± 7.1	22.6 ± 24.4	52.3 ± 20.4	95.7	3	< 0.001*	CG > BP-ED > AN-R

*BMI* body mass index; *AN-R* anorexia nervosa, restricting type; *BP-ED* binge-purging eating disorders [BN (bulimia nervosa) + AN-BP (anorexia nervosa, binge-purging type)]; *CG* healthy control group

*p < 0.05

**Table 2:** Sample characteristics: psychiatric comorbidities

	Group	
	AN-R (*n* = 54)	BP-ED (*n* = 33)
	*n* (%)	*n* (%)
At least one comorbidity	19 (35.2)	14 (42.4)
Type of comorbidity		
Mood disorders (F30-F39):	13 (24.1)	22 (66.7)
F32.0 Depressive episode, mild	2 (3.7)	1 (3.0)
F32.1 Depressive episode, moderate	6 (11.1)	4 (12.1)
F33.1 Depressive episode, relapsing, moderate	1 (1.9)	0 (0.0)
F34.1 Dysthymia	3 (5.6)	3 (9.1)
F34.8 Other persistent affective disorders	1 (1.9)	4 (12.1)
Neurotic, stress and somatoform disorders (F40-F49):	5 (9.3)	2 (6.1)
F40.1 Social phobia	0 (0.0)	1 (3.0)
F40.2 Specific phobia	0 (0.0)	1 (3.0)
F42.0 Obsessive disorder, predominantly obsessional thoughts	1 (1.9)	0 (0.0)
F42.1 Obsessive disorder, predominantly obsessional acts	2 (3.7)	0 (0.0)
F43.1 Post-traumatic stress disorder	1 (1.9)	0 (0.0)
F45.4 Persistent pain disorder	1 (1.9)	0 (0.0)
Other	1 (1.9)	0(0.0)

*AN-R* anorexia nervosa, restricting type; *BP-ED* binge-purge eating disorders [BN (bulimia nervosa) + AN-BP (anorexia nervosa, binge-purging type)

Age differed significantly between the groups [*F*(3,125), *p* = 0.025], with the BP-ED having the oldest participants. Furthermore, the ED groups significantly differed with regard to their BMI [*F*(3,125) = 102.5, *p* < 0.001] and BMI percentiles [*F*(3,125) = 95.7, *p* < 0.001], with AN-R patients scoring the lowest (Table 1). Groups differed significantly in the prevalence of comorbidities [*χ*^*2*^(3) = 24.0, *p* < 0.001] with BP-ED patients having the most comorbidities [BP-ED: 42.4% (*n* = 14), AN-R: 35.2% (*n* = 19)]. Regardless of group, mood disorders were the most frequent comorbidities [BP-ED: 36.4% (*n* = 12), AN-R: 24.1% (*n* = 13)].

A two-way factorial MANOVA was conducted with the group (AN-R, BP-ED, CG) as fixed factors, CEN and CEA as covariates, and conscientious-compulsive and spontaneous-borderline as dependent variables. Descriptive statistics are shown in Table 3. The evaluation of the homogeneity of the variance-covariance matrices and normality assumptions underlying MANOVA did not reveal any substantial anomalies.

**Table 3:** Descriptive statistics for childhood emotional neglect, childhood emotional abuse, conscientious-compulsive and spontaneous-borderline personality styles

	AN-R		BP-ED		CG	
	*N*	*M* ± SD	*N*	*M* ± SD	*N*	*M* ± SD
CEN	54	9.4 ± 3.2	33	9.9 ± 3.5	41	8.2 ± 2.8
CEA	54	8.7 ± 4.6	33	9.3 ± 4.0	41	6.2 ± 2.0
CO	54	15.4 ± 5.6	33	13.6 ± 4.7	41	14.4 ± 5.0
BL	54	9.9 ± 5.8	33	14.2 ± 6.2	41	7.6 ± 5.5

*Raw values of the variables CEN = childhood emotional neglect, CEA*  childhood emotional abuse, *CO*  conscientious-compulsive, *BL*  spontaneous-borderline for the groups, *AN-R* anorexia nervosa, restricting type, *BP-ED* binge-purging eating disorders [BN (bulimia nervosa) + AN-BP (anorexia nervosa, binge-purging type)], *CG* healthy control group

**Fig. 1:**
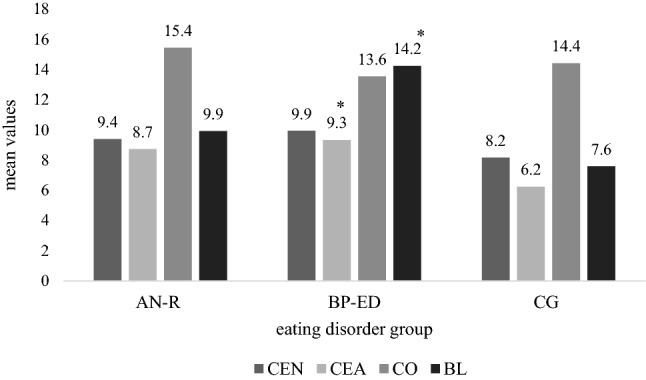
Childhood emotional neglect, childhood emotional abuse, conscientious-compulsive and spontaneous-borderline personality styles across eating disorder types. Note. *CEN* childhood emotional neglect, *CEA* childhood emotional abuse, *CO* conscientious-compulsive, *BL* spontaneous-borderline for the groups, *AN-R* anorexia nervosa, restricting type, *BP-ED* binge-purging eating disorders [BN (bulimia nervosa)+ AN-BP (anorexia nervosa, binge-purging type)], *CG* healthy control group, *p < 0.05

There was a significant main effect of CEA on spontaneous-borderline [*F*(8,119) = 17.1, *p* < 0.001, *η*^*2*^ = 0.126] as well as a significant main effect of ED group on spontaneous-borderline [*F*(2,119) = 3.1, *p* = 0.048, *η*^*2*^ = 0.050]. The MANOVA revealed a significant interaction between ED group, CEA and spontaneous-borderline, *F*(2,119) = 3.5, *p* = 0.034, partial *η*^*2*^ = 0.055, with BP-ED showing significantly higher scores in CEA (9.9 ± 3.5) and spontaneous-borderline (14.2 ± 6.2) (Table 4). No further interaction effects were found. No main effect of CEN on conscientious-compulsive or spontaneous-borderline was found. Figure 1 displays the main effects and the interaction effect.

**Table 4:** MANOVA for childhood emotional neglect and childhood emotional abuse on conscientious-compulsive and spontaneous-borderline personality styles in patients with eating disorders

	MANOVA							
	CO				BL			
	*F*	*df*	*p*	*η*^*2*^	*F*	*df*	*p*	*η*^*2*^
ED diagnosis	1.4	2	0.251	0.02	2.9	2	0.060	0.05
CEN	0.1	1	0.826	0.00	2.2	1	0.138	0.02
CEA	0.1	1	0.745	0.00	17.0	1	< 0.001*	0.12
ED diagnosis*CEN	1.5	2	0.235	0.03	0.5	2	0.603	0.01
ED diagnosis*CEA	1.1	2	0.348	0.02	3.3	2	0.042*	0.05

*ED*  eating disorder, *CEN*  childhood emotional neglect, *CEA* childhood emotional abuse, *CO* conscientious-compulsive, *BL* spontaneous-borderline

*p < 0.05

## Discussion

The aim of the present study was to investigate the interaction between CEN, CEA, and obsessive-compulsive and borderline personality styles in adolescents with BP-ED and AN-R compared to a healthy CG. Our results revealed a significant interaction between CEA and borderline personality style in BP-ED patients. This finding is consistent with previous results showing an association between BN and borderline PD in adolescent and adult patients [26]. Furthermore, the present findings are in accordance with prior studies suggesting a relationship between borderline PD and CEA in adults [16, 31]. CEA, borderline PD, and BP-ED have been associated with difficulties in emotion regulation [30, 31]. Thus, one possible explanation for our results is that emotionally abused patients develop impulsive behaviors such as binge eating and purging, serving as dysfunctional emotion regulation strategies to modulate the negative emotional effects of abuse. Our results support previous research indicating that exposure to an invalidating, traumatic environment such as CEA may play a role in the development and maintenance of borderline personality styles and BP-ED. However, longitudinal studies are necessary to investigate the direction of this potential association. In addition, studies are needed to evaluate the relationship between binge eating and purging behaviors, and emotion regulation.

Contrary to our hypothesis, we did not find an association between CEN, CEA, and obsessive-compulsive personality style in AN-R patients. Adolescent AN-R patients who had experienced CEA or CEN did not appear to develop compulsive behaviors as an emotion regulation strategy. However, our results are in contrast to reports by previous studies [15–17, 44]. These inconsistencies may be due to methodological differences, such as the investigation of personality style vs. PD or the focus on CEA and CEN vs. sexual and physical abuse. Furthermore, our study focused on adolescents, while previous studies had only included adult patients. Moreover, denial and minimization of psychological problems appear to be more common in AN than in BN, especially in patients with AN-R, as previously indicated by various studies [45–47]. Therefore, it is also possible that patients with AN-R scored lower on the CTQ subscales because they had denied traumatic experiences in childhood.

The findings of this study should be interpreted in the context of several limitations. First, the sample size was modest, especially the BP-ED subgroup. Second, CEN and CEA were assessed retrospectively using a self-report questionnaire and were not examined by a clinical expert-interview. No information about the intensity, duration, or time of CEN and CEA was gathered. While retrospective self-reports of CEN and CEA are a standardized, accepted, and widely used procedure in clinical practice [48, 49], further evaluation by a clinical expert could help achieve more accurate results. Third, our results are limited by the cross-sectional study design. In future research, longitudinal studies with larger sample sizes should be conducted to examine the causality of CEA, CEN, EDs, and personality styles. Despite these limitations, this is, to our knowledge, the first study examining the interaction between CEN, CEA, and specific personality styles in adolescents with AN and BN.

As childhood and adolescence represent a crucial phase in personality development [33, 34], further studies should focus on these specific age groups. An early assessment of predisposing factors may contribute to a better understanding of the etiology and maintenance factors of EDs [24] as well as provide leads for additional or alternative treatment approaches.

In summary, our results suggest that CEA and borderline personality styles are of particular importance in female adolescents with BP-ED. Further research on the interaction between CEA and personality styles in adolescents with EDs is needed, specifically longitudinal studies to investigate the causality. Considering CEA and borderline personality styles in adolescent patients with BP-ED may help improve the understanding of the etiology and maintenance of BP-ED and to provide leads for more effective treatments. Clinicians should be sensitized to the interaction of CEA and borderline personality styles in patients with BP-ED in both clinical diagnostics and treatment. Standardized assessment of trauma exposure as well as additional treatment interventions for those patients with CEA and borderline personality styles, such as enhancing emotion regulation strategies and/or trauma treatment, may improve treatment outcomes in this patient group.

## What is already known on this subject?

- Adults with an eating disorder often report a history of childhood sexual or physical abuse and suffer from comorbid borderline personality disorder. Little is known about childhood emotional abuse and personality styles in adolescents with eating disorders.

## What does this study add?

- Childhood emotional abuse is associated with spontaneous-borderline personality style in adolescent inpatients with binge-purging eating disorders (anorexia nervosa, binge-purging type and bulimia nervosa).
